# Preparation and In Vitro Evaluation of a Gadolinium-Containing Vitamin E TPGS Micelle as a Potential Contrast Agent for MR Imaging

**DOI:** 10.3390/pharmaceutics15020401

**Published:** 2023-01-25

**Authors:** Yongkang Gai, Yuying Li, Shuangping Wu, Ling Xu, Yao Lu, Xiaoli Lan, Guangya Xiang, Xiang Ma

**Affiliations:** 1 School of Pharmacy, Tongji Medical College, Huazhong University of Science and Technology, 13 Hangkong Road, Wuhan 430030, China; 2 Department of Nuclear Medicine, Union Hospital, Tongji Medical College, Huazhong University of Science and Technology, Wuhan 430022, China; 3 Hubei Province Key Laboratory of Molecular Imaging, Wuhan 430022, China; 4 School of Pharmacy, Tongren Polytechnic College, Tongren 554300, China

**Keywords:** MRI contrast agents, micelle, TPGS, gadolinium

## Abstract

The application of many currently evaluated macromolecular contrast agents for magnetic resonance imaging (MRI) has been limited because of their bio-incompatibility and toxicity. The aim of this study is to synthesize and characterize a new micelle-based TPGS gadolinium chelate as a biocompatible MRI contrast agent for prolonged blood circulation time and good tumor imaging contrast. The TPGS-gadolinium conjugate was prepared through the conjugation between TPGS-SA and bifunctional L-NETA-Gd chelate. The conjugate was characterized with regard to molecular weight, critical micellar concentration and particle sizes, cellular uptake, and in vitro cell MRI. Distributions of the MRI contrast agent in various organs were determined via intravenous injection of the agent into mice bearing xenograft tumors. The successfully prepared TPGS-L-NETA-Gd micelle exhibited improved cellular uptake in HepG2 cells and xenografts and high in vivo safety. Distributions of TPGS-L-NETA-Gd in mice showed enhanced cellular uptake up to 2 h after the contrast agent injection. Its in vitro and in vivo properties make it a favorable macromolecular MRI contrast agent for future in vivo imaging.

## 1. Introduction

**Figure 1:**
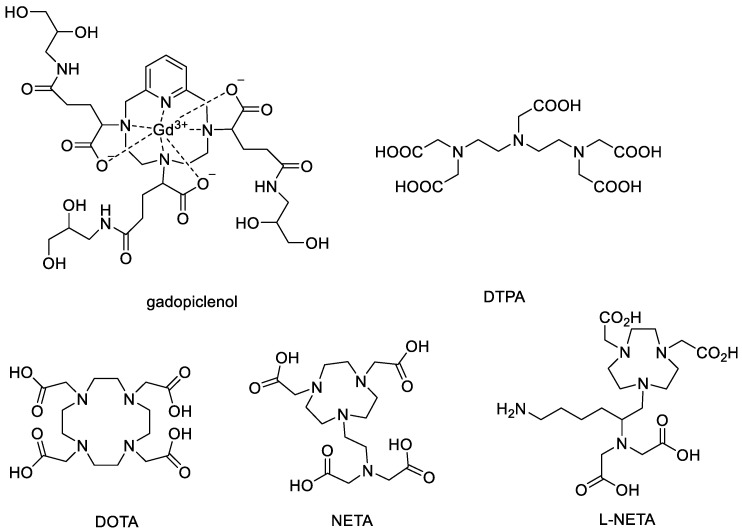
Structures of gadopiclenol and some gadolinium chelators for MRI imaging.

Nowadays, various imaging technologies, such as ultrasound, computed tomography (CT), magnetic resonance imaging (MRI), positron emission tomography (PET), and single-photon emission computed tomography (SPECT), are used for the evaluations of diagnosis and therapy [1,2]. Among them, MRI, a routinely used imaging modality effective for both anatomical and functional imaging of diseased soft tissues, including solid tumors, offers many advantages owing to its high spatial and temporal resolution [3]. Contrast agents (CAs) are frequently used to enhance the intrinsic MR signal, with Gd (III) complexes being the most commonly used agents in clinic [4]. To date, ten different types of small-molecular-weight gadolinium-containing CAs are available in different territories, including gadopiclenol, which was approved by the FDA (Elucirem™, VUEWAY™) in September 2022 (Figure 1). Gadopiclenol is a nonionic macrocyclic CA with high thermodynamic and kinetic stabilities, and the highest r1 relaxivity among currently available CAs. However, most contrast agents in clinical settings have the nonspecific extracellular distribution property and the disadvantages of low relaxivity, low tissue specificity, and rapid clearance. Thus, many efforts have been made to develop better CAs with high relaxivity, low toxicity, and high tumor specificity [3,4]. Accordingly, in order to optimize their pharmacokinetic properties for experimental and preclinical detection of solid tumors, various nanoparticles have been considered as delivery carriers for CAs, such as polymeric micelles, liposomes, polymersomes, iron oxide nanoparticles, lipid nanoparticles, and other platforms [5,6,7].

Current CAs generate positive image contrast by decreasing the proton spin–lattice relaxation time (T1) of water protons [8]. Gadolinium (Gd)-based compounds, such as gadopentetate dimeglumine (Gd-DTPA, Magnevist^TM^), have been utilized preclinically for cell labeling in order to enable tracking and monitoring of stroke lesions or tumor growth in vivo [9]. However, Gd-DTPA has a blood half-time of less than 5 min, which is insufficient to enhance the contrast of tumor tissues [3]. In recent years, micelles have attracted much attention as targeted CA carriers [10,11]. Micelles are amphiphilic spherical nanostructures consisting of a hydrophobic core and a hydrophilic shell. Polymeric micelles with amphiphilic copolymers usually exhibit very low critical micelle concentration (CMC) and are more stable than micelles formed from surfactants [12]. They cannot be easily eliminated by the reticuloendothelial system due to their small dimension (diameter ≤ 50 nm) and their hydrophilic shell, thus showing an enhanced permeability and retention effect (EPR effect) [13]. Micellar Cas could be easily obtained by grafting gadolinium chelates to the hydrophilic layer of polymer micelles to achieve increased relaxivity and long-circulating properties [11]. The choice of amphiphilic materials is one of the most crucial factors for the construction of stable and safe micelles [14]. D-a-Tocopherol polyethylene glycol 1000 succinate (TPGS), a water-soluble derivative of natural vitamin E and polyethylene glycol 1000, is approved as a safe pharmaceutical adjuvant by the FDA [15,16]. As a safe nonionic surfactant, it exhibits amphipathic properties and can form stable micelles in aqueous vehicles. TPGS has been widely investigated for the delivery of chemo-drugs or imaging agents [17,18]. Therefore, the use of TPGS as part of the ingredients in a new micelle-based MRI contrast agent is a promising alternative since it is possible to combine its safety profile with biocompatible and pharmacotechnical properties.

The influence of a metal chelator that coordinates gadolinium is another important factor for the construction of MRI CAs. The acyclic chelator acyclic DTPA (diethylenetriamine-N,N,N′,N′,N″-pentaacetic acid) and the macrocyclic DOTA (1,4,7,10-tetraazacyclododecane-1,4,7,10-tetraacetic acid) systems and their derivatives are among the most commonly used chelators in experimental and preclinical studies (Figure 1). The application of DOTA as a chelator for gadolinium is largely due to its ability to form complexes with high thermodynamic stability and kinetic inertness [19]. However, slow complex formation kinetics with DOTA under mild conditions is a major disadvantage. The acyclic octadentate DTPA and its bifunctional versions form complexes much faster than DOTA derivatives, but these complexes have significantly lower kinetic inertness than the corresponding DOTA derivatives [20]. Stable Gd (III) complexes with low kinetic inertness are required for the safe use of CA agents since transmetallation-freed Gd (III) is toxic and is known to cause severe side effects [21]. Considerable research efforts have been directed toward developing safe Gd (III)-based MR contrast agents; the 1,4,7-triazacyclonane (TACN)-based chelator NETA and its derivatives attracted our attention due to their unique structures that integrate the advantages of both macrocyclic and acyclic frameworks for favorable thermodynamic stability and chelating kinetics [22,23,24,25,26]. In addition, when coordinated with other metals, including ^64^Cu, ^68^Ga, Al^18^F, and ^177^Lu, these TACN-based chelators reported in our group could also be used as PET/SPECT tracers and/or therapeutic agents for various medical applications [24,27,28].

Among these reported ligands, our previously developed bifunctional chelator L-NETA [29,30] (Figure 1), which possesses both TACN and acyclic lysine moieties for cooperative metal binding, is proposed to rapidly initiate coordination to Gd (III) and is expected to achieve the maximum complex stability with the metal. Its potential high relaxivity, mild Gd (III) chelating conditions, and high stability also make it attractive as a potential gadolinium chelator for the construction of MRI CAs. Herein, we aimed to develop a new micelle-based MRI contrast agent with the bifunctional chelator L-NETA as the fundamental material for prolonged blood circulation time and better tumor imaging contrast with TPGS. After the formulation, the TPGS-L-NETA-Gd micelles (denoted as TLNm) were characterized and evaluated via a series of in vitro and in vivo experiments. Ex vivo gadolinium biodistribution was also carried out to investigate the in vivo performance of the prepared micelle in HepG2 tumor-bearing mice.

## 2. Experimental Section

### 2.1. Materials

L-NETA was previously synthesized in our lab (in-house chemical) [29,30]. D-a-Tocopherol polyethylene glycol 1000 succinate (TPGS) and succinic anhydride were obtained from Sigma-Aldrich (St. Louis, MO, USA). Gd-DTPA (Magnevist) was purchased from Bayer (Leverkusen, Germany). Fetal bovine serum (FBS) was obtained from Zhejiang Tianhang Biological Technology Co., Ltd. (Hangzhou, China). DMEM were purchased from Sigma-Aldrich Corp. (St. Louis, MO, USA). HCC cell line HepG2 was obtained from the China Center for Type Culture Collection at Wuhan University (Wuhan, China). BALB/c nude, female (20 g, 6–8 weeks old) mice were purchased from Beijing Huafukang Bioscience Technology Co., Ltd. (Beijing, China).

### 2.2. Synthesis of TPGS-SA

TPGS was first modified with succinic anhydride (SA) to obtain TPGS-SA with a free terminal carboxylic acid functional group. Briefly, to a solution of 1.5 g of TPGS (1 mmol) in 20 mL of dichloromethane (DCM), 0.2 g of SA (1.9 mmol) and 0.12 g of 4-dimethylaminopyridine (DMAP, 1 mmol) were added, and the resulting mixture was reacted for 24 h at room temperature. The crude product was precipitated in cold diethyl ether and then purified using silica column chromatography (gradient: from 5% MeOH in DCM to 10% MeOH in DCM).

### 2.3. Synthesis of L-NETA-Gd

Gadolinium oxide (0.68 g, 1.9 mmol) was added to a solution of 1 g of bifunctional chelator L-NETA (2 mmol) in 5 mL of DI water. The reaction mixture was heated to 100 °C and reacted for 6 h. Over that time period, the solid oxide dissolved and the pH increased from 2 to around 7. The xylenol orange test was applied to determine the presence of free Gd^3+^ during the reaction. After confirming the absence of free Gd^3+^, the reaction mixture was lyophilized to obtain L-NETA-Gd as a white powder. MALDI-HRMS (matrix: HCCA): *m/z* calcd for C_20_H_34_GdN_5_O_8_ [M+H]^+^: 630.1648; Found 631.1720.

### 2.4. Synthesis of TPGS-L-NETA-Gd

TPGS-SA (0.8 g, 0.5 mmol), dicyclohexylcarbodiimide (DCC, 0.2 g, 1 mmol), and DMAP (0.1 g, 0.8 mmol) were dissolved in 10 mL of dimethyl sulfoxide (DMSO), and the resulting mixture was incubated at room temperature for 1 h. L-NETA-Gd (0.2 g, 0.55 mmol) and triethylamine (TEA) (0.2 g, 2 mmol) were added to the reaction mixture and further incubated for 20 h at room temperature. The reaction mixture was filtered to remove N,*N*-dicyclohexylurea (DCU) and then dialyzed using MWCO 500 membrane in water for 24 h to remove DMSO and unconjugated L-NETA-Gd. The resulting solution was lyophilized to obtain TPGS-L-NETA-Gd as a waxy solid. Estimated mass: 2300 ± 500; Found: 2300.1999 ± 200 (MALDI-HRMS, matrix: HCCA).

### 2.5. Preparation of TLNm

To prepare TLNm, TPGS-L-NETA-Gd was dissolved in CHCl_3_ and dried to a thin film in a round-bottomed flask on a rotary evaporator under reduced pressure for 30 min at 45 °C. After it was evaporated by rotary evaporation, the dried lipid mixture was then rehydrated in 2 mL of phosphate buffer (pH 7.4) at 60 °C for 1 h under flowing nitrogen. The resulting suspension of vesicles was extruded through the 200 nm and 100 nm pore size polycarbonate membranes at least five times using a Lipex extruder (Northern Lipids Inc., Vancouver, BC, Canada).

### 2.6. Cell Culture and Animal Tumor Model

HepG2 and LO2 cells were cultured with high-glucose DMEM supplemented with penicillin, streptomycin, and 10% FBS in a 37 °C and 5% CO_2_ incubator.

BALB/c nude, female (20 g, 6 weeks old) mice and Kunming mice were purchased from Beijing Huafukang Bioscience Technology Co., Ltd. (Beijing, China). The animals were cared for under an animal use protocol approved by Huazhong University of Science and Technology. The study was approved and conducted according to the rules set forth by the Institutional Animal Care and Use Committee (IACUC) of Tongji Medical College, Huazhong University of Science and Technology (IACUC No. S2648, January 2021).

To induce a tumor, HepG2 cells (2.5 × 10^6^ cells per mouse) were suspended in 100 µL of PBS and injected subcutaneously into BALB/c nude mice. When the tumor size reached about 5 mm in diameter after about 10 days, the nude mice were used for the in vivo study.

### 2.7. Transmission Electron Microscopy (TEM) Measurements

A drop of the sample solution was placed on a 400-mesh copper grid. After the 30 min deposition, the solution was removed by using filter paper and then negatively stained with 1% phosphorus acid. The shape of TLNm was determined on a transmission electron microscope (JEOL 100CX II TEM, Tokyo, Japan) at an accelerating voltage of 100 kV.

### 2.8. Determination of the Critical Micellar Concentration (CMC)

The CMC of micelles in water was determined with a fluorospectrometer using pyrene as a hydrophobic fluorescence probe [5]. Briefly, a 1 mg/mL solution of T-L-Gd was prepared in DCM. Different volumes of this solution were added to 20 mL empty vials. Then, 50 μL of 1.8 × 10^−4^ M solution of pyrene in DCM was added in every vial and mixed well. The DCM was left to evaporate for 24 h in order to form a pyrene film in the vial. Finally, 15 mL of Milli-Q water was added to the vials to obtain a final pyrene concentration of 6.0 × 10^−7^ M for each vial and T-L-Gd solutions with concentrations ranging from 0.0002 mg/mL to 0.2 mg/mL. The solutions were kept on a shaker at 37 °C for 24 h to reach equilibrium before the fluorescence measurement. Fluorescence spectra were recorded on a luminescence spectrometer at room temperature. The excitation spectra were scanned from 300 to 350 nm at the emission wavelength of 373 nm. Excitation and emission bandwidths were 5 nm and 10 nm, respectively. The fluorescence intensity ratio of I342/I338 was analyzed as a function of micelle concentration.

### 2.9. Uptake of TLNm

To analyze the uptake of TLNm and Gd-DTPA in vitro, HepG2 cells were plated on 24-well plates at a density of 1 × 10^5^ cells/well and incubated for 24 h with high-glucose DMEM supplemented with penicillin, streptomycin, and 10% FBS in a 37 °C and 5% CO_2_ incubator. The concentrations of TLNm and Gd-DTPA were diluted into 20 μg/mL (with Gd content) and were added to the medium for 8 h, 4 h, 2 h, 1 h, or 30 min. In order to measure the uptake of TLNm and Gd-DTPA, cells were washed with PBS 3 times and then trypsinized, collected, and digested in aqua regia. The concentration of the Gd (III) was determined via ICP-AES (Prodigy 7, LEEMAN LABS Ltd., Hudson, NY, USA).

### 2.10. In Vitro Cell MRI

For the cellular MRI, HepG2 cells were seeded into culture dishes with 10 mL of culture medium. When the cells reached 80% confluence, the medium was replaced with fresh medium containing different samples (TPGS-NETA-Gd micelles and Gd-DTPA at the same gadolinium concentration of 20 μg/mL). Meanwhile, another dish replaced with only fresh medium was selected as a control. After 2 h of incubation, the medium was removed and washed with PBS three times. Then, cells were harvested after the treatment with trypsin and centrifugation. The resulting cells were transferred to 200 μL Eppendorf (EP) tubes and then centrifuged at 1000 rpm to obtain a compact pellet at the bottom of the tube and directly measured for MRI. In the in vitro experiments, HepG2 cells or LO2 cells were grown in different concentrations of TLNm and Gd-DTPA complex (final concentrations of 20, 40, or 80 μg/mL diluted in Dulbecco’s minimal essential medium (DMEM) supplemented with 10% fetal bovine serum FBS) or PBS, in 1.5 mL EP tubes (2 × 10^6^ cells /tube). The cells were subsequently incubated for 2 h at 37 °C with 5% CO_2_. Cells in each EP tube were transferred at 1500 rpm and washed with PBS (3 × 1 mL). Cells were resuspended with 500 μL of 1 × PBS (PH = 7.4). A multi-slice SE sequence was used.

### 2.11. Acute Toxicity Studies of TLNm

Acute toxicity of TLNm was evaluated based on histological observations of organs and plasma protein levels. Pathologic examinations of livers and kidneys were conducted 5 days after tail vein injections of TLNm (0.1 mmol Gd/kg). Livers and kidneys were dissected, fixed in 4% neutral paraformaldehyde solution, embedded in paraffin wax, and further sliced and stained with hematoxylin and eosin (H&E) for microscopic observation.

TLNm was injected into Kunming mice at 0.1 mmol Gd/kg and, 5 days later, blood was sampled by cardiac puncture and collected in EDTA tubes. Plasma samples were separated by centrifugation at 2000× *g* for 10 min. Microplate assay was used for the determination of aspartate aminotransferase (AST) and alanine aminotransferase (ALT).

### 2.12. Biodistribution

A xenograft mouse model was generated by subcutaneous injection of HepG2 cells (2.5 × 10^6^ cells per mouse) into the right hind flank of BALB/c female mice. The biodistribution of TLNm was determined at 0.5 h, 1 h, 2 h, and 4 h post-injection in comparison with Gd-DTPA. The contrast agents were administered at a dose of 0.1 mmol Gd /kg (TLNm concentration of 290 μg/mL) via tail vein injection. ICP-AES was used to measure the content of gadolinium in organs. Before the analysis, organs were dehydrated via freeze-drying. Tissue samples were crushed, then digested and mineralized with aqua regia (1 mL for each organ) at room temperature for 48 h. Finally, the solution was fixed in a 2 mL capacity bottle.

### 2.13. Statistical Analysis

The comparison of two groups was performed using Student’s *t*-test (SPSS Software, Chicago, IL, USA). Multiple groups were compared using a one-way ANOVA with Dunnett’s post-test. A value of *p* < 0.05 was considered significant and *p* < 0.01 was considered highly significant.

## 3. Results and Discussion

### 3.1. Synthesis and Characterization of TPGS-L-NETA-Gd

**Figure 2:**
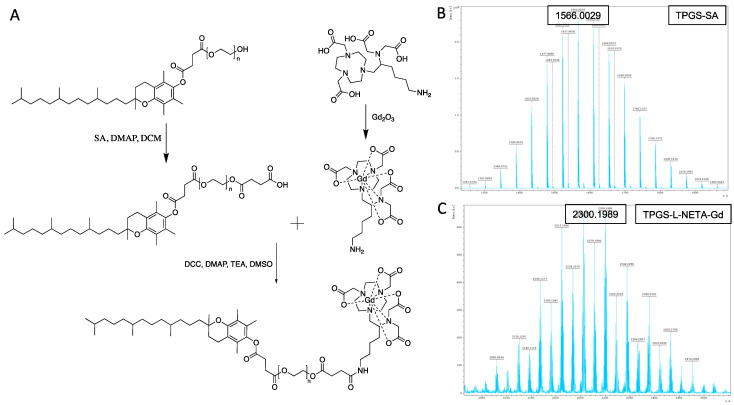
(**A**) Synthesis of L-NETA-Gd and TPGS-L-NETA-Gd (T-L-Gd); (**B**) mass spectrum of TPGS-SA; (**C**) mass spectrum of TPGS-L-NETA-Gd.

The synthesis of TPGS-L-NETA-Gd was achieved smoothly via a multistep synthesis (Figure 2A). To anchor Gd (III) efficiently and stably, a bifunctional chelator L-NETA developed previously by our group was used. While chelating, a small amount of excess L-NETA was applied to ensure a quantitative chelation of GdCl_3_. The absence of free Gd^3+^ was confirmed by the xylenol orange test. A solid white powder was formed after the lyophilization. The MALDI-TOF mass results with multiple peaks of the isotopes of gadolinium confirmed that L-NETA-Gd was formed. In order to conjugate TPGS with L-NETA-Gd, a succinic acid linker was introduced to TPGS. With the amide bond formation between TPGS-SA and L-NETA-Gd, TPGS-L-NETA-Gd was successfully prepared and purified using dialysis to remove the salt and unreacted L-NETA-Gd. The masses of TPGS-SA and TPGS-L-NETA-Gd were tested using MALDI-TOF, and the spectra shown in Figure 2B,C, respectively, validate the successful synthesis of TPGS-L-NETA-Gd material.

### 3.2. Determination of the CMC and Particle Sizes

**Figure 3:**
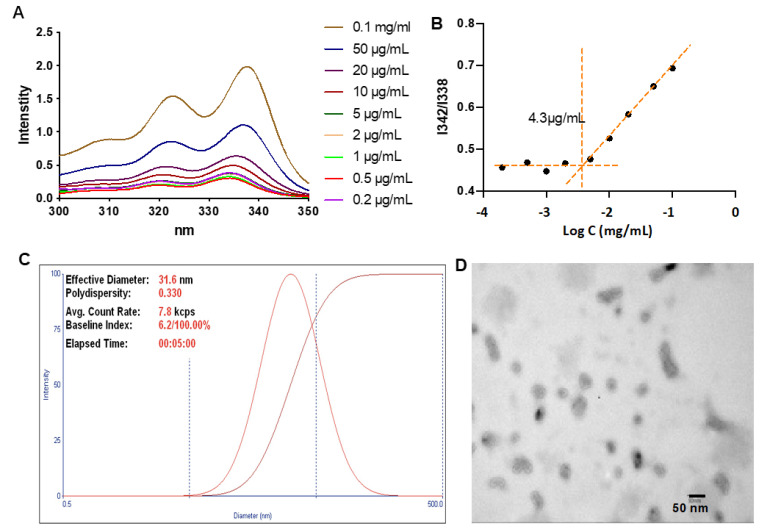
(**A**) Determine the CMC of TPGS-L-NETA-Gd; (**B**) the value of CMC was 4.3 µg/mL; (**C**) DLS data of TLNm in water; (**D**) TEM bright-field image of TLNm dried on formvar-coated copper grids (scale bar = 50 nm).

CMC is a vital property of micelles, affecting their stability and drug-loading efficiency for drug delivery. Thus, before further evaluation, the CMC of TLNm was determined using a traditional method based on a hydrophobic fluorescence probe pyrene [31]. As shown in Figure 3A,B, the ratios of I342/I338 were nearly unchanged at low concentrations of TPGS-L-NETA-Gd, whereas the ratios decreased at higher concentrations, indicating the self-aggregation of TPGS-L-NETA-Gd. The CMC value of TLNm was determined to be 4.3 µg/mL, which is comparable to that of TPGS, indicating the modification of TPGS did not significantly affect its ability to form micelles.

After determining the CMC, the TPGS-L-NETA-Gd micelle (denoted as TLNm) was subsequently diluted in PBS and passed through a 0.22 filter. The particle size distribution of TLNm was characterized by a dynamic light-scattering (DLS) technique. As shown in Figure 3C, the diameter was around 31.6 nm with a polydispersity of 0.33. The spherical morphology and mono-dispersity of TLNm were also confirmed by TEM, and the size was measured to be nearly 25 nm (Figure 3D), which is consistent with the results of DLS.

### 3.3. Uptake of TLNm

**Figure 4:**
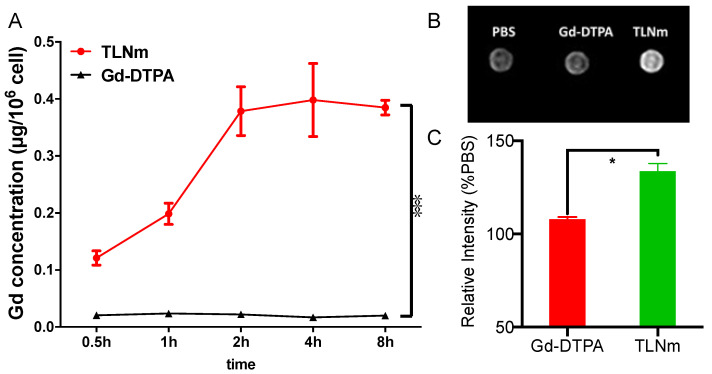
(**A**) Cell uptake of TLNm (red) and Gd-DTPA (black) was determined in HepG2 cells and showed a highly significant difference (***, *p* < 0.001; *n* = 3); (**B**) T_I_-weighted axial scan for HepG2 cells; (**C**) the signal intensity ratio of HepG2 cells treated with PBS, Gd-DTPA, and TLNm. Cells incubated with samples at a Gd concentration of 20µg/mL for 2 h. Signal intensity showed a statistically significant difference (*, *p* < 0.05; *n* = 3). Notes: Here, PBS served as a blank control without Gd. Abbreviations: TLNm, TLNm; Gd-DTPA, gadopentetate dimeglumine, Magnevist.

To determine the cell uptake property of TLNm, HepG2 cells were used, and the results were compared with those of Gd-DTPA (Figure 4A). The uptake of TLNm increased with time in the first 2 h, then reached a plateau at 2 h, and remained at the same level up to 8 h. Significantly higher uptake than that of the Gd-DTPA group was observed in TLNm groups at all examined time points. As expected, Ga-DTPA uptake into the cells was low. The introduction of TPGS significantly increased the cellular uptake of gadolinium.

### 3.4. In Vitro Cell MR

To investigate the ability of micelles to enhance T1-weighted contrast of cell populations, MR images (shown in Figure 4B) and the resulting signal intensity ratio of HepG2 cell pellets were acquired via a 3 T human MR scanner. HepG2 cells were incubated with TLNm, Gd-DTPA, and PBS, respectively. In comparison with cells incubated with PBS, cells with Gd-DTPA presented no or a slight improvement in contrast. However, the cells incubated with TLNm showed obviously enhanced contrast, which could be attributed to the function of TPGS. Moreover, the intensity of signal was measured to quantitatively analyze the difference in MR images (shown in Figure 4C). The PBS group was set as a baseline, and the signal intensity of Gd-DTPA-treated cells was slightly improved to 106 ± 2%, whereas that of cells incubated with TLNm was increased to 137 ± 3%.

### 3.5. In Vivo Biodistribution

**Figure 5:**
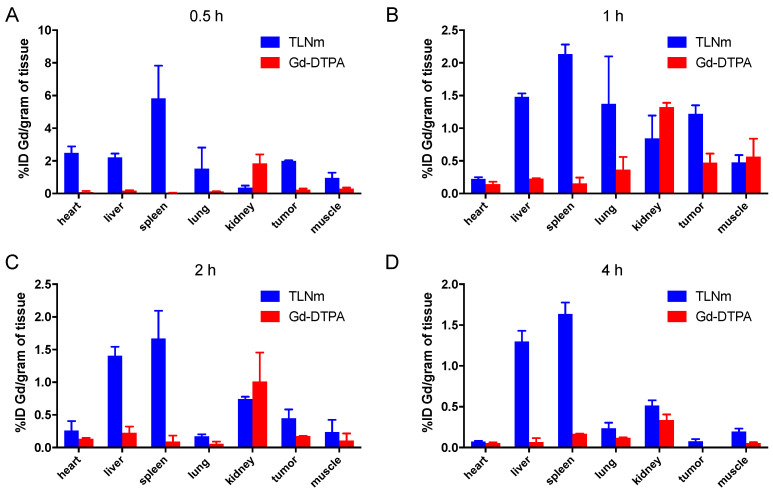
Ex vivo gadolinium biodistribution in HepG2 tumor-bearing mice at 0.5 h (**A**), 1 h (**B**), 2 h (**C**), and 4 h (**D**) post-injection of Gd-DTPA and TLNm, respectively (dose: 0.1 mol Gd/kg).

To determine whether TLNm can be delivered into a tumor in vivo, we examined the distribution of TLNm in HepG2 tumor-bearing nude mice. The animals were sacrificed at different time points after the injection of TLNm and Gd-DTPA, respectively, and the amounts of gadolinium in tissues were analyzed using ICP-AES. As shown in Figure 5, the data illustrate that TLNm displayed higher gadolinium levels in all of the organs, except for the kidney, at all time points. TLNm displayed relatively slow clearance from the kidney. At 1 h, tumor uptake of the complex reached the highest level, at 1.3 % ID/g. The Gd-DTPA complex was essentially extracellular in vivo and resulted in rapid blood clearance and very low organ uptake (Figure 5). At 1 h and 2 h, the kidney uptake levels were at 1.0–1.5% ID/g. At 1 h, the ratios of tumor to liver and tumor to spleen were 0.9 and 0.6, respectively. The high uptake of TLNm in the liver and spleen was noteworthy in that TPGS was found to be liver-targeting. Higher tumor uptake of TLNm than of Gd-DTPA was observed throughout all time points. These in vitro biodistribution data confirmed that the tumor- and liver-targeting performance of TLNm was higher than that of Gd-DTPA. This study has fulfilled our purpose and also exhibited the maximum effect on tumor uptake and duration of time compared to the control.

### 3.6. Acute Toxicity Studies of TLNm

**Figure 6:**
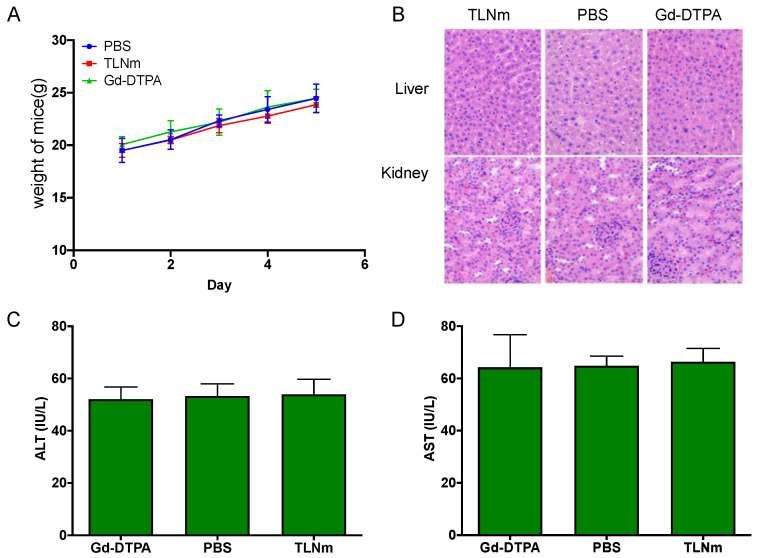
The weight change of KM mice five days after an injection of TLNm, Gd-DTPA at a dose of 0.1 mmol Gd/kg, and PBS (**A**). Tissues were recovered 5 days after an injection of TLNm, Gd-DTPA at a dose of 0.1 mmol Gd/kg, and PBS. H&E-stained liver and kidney tissues (magnification, 200×) (**B**). The values of ALT (**C**). The values of AST (**D**).

To further investigate the in vivo safety of TLNm, Kunming mice were executed 5 days after the injection of TLNm, Gd-DTPA, and PBS (control group), respectively. There were no significant inflammatory lesions or tissue damages in major organs (liver and kidney) compared to the control group. There was also no significant difference in weight changes among the three groups (Figure 6A), and the values of ALT and AST showed no significant difference in the three groups, indicating that the synthesized TLNm has good biocompatibility.

## 4. Conclusions

In the current study, a gadolinium-containing micelle based on an FDA-approved material, vitamin E TPGS, was prepared using L-NETA as a chelating agent. The preparation and characteristics of the TPGS-L-NETA-Gd micelles were studied successfully. The CMC value of TLNm was determined to be 4.3 µg/mL, which is comparable to that of TPGS. The spherical morphology and monodispersity of TLNm were also confirmed via TEM, and the size was measured to be nearly 25 nm. In vitro cell-based assays confirmed the introduction of TPGS significantly increased the cellular uptake of gadolinium and showed obviously enhanced contrast compared to Gd-DTPA. The prepared TPGS-L-NETA-Gd micelles also exhibited high in vivo safety, and improved uptake in HepG2 cells and xenografts, rendering them potential MR contrasting agents for enhanced tumor imaging.

## Data Availability

The data presented in this study are available on the request to the corresponding authors.
